# Supplementary material for: Individualized machine-learning-based clinical assessment recommendation system
Source: PLOS Digit Health. 2025 Sep 25;4(9):e0001022. doi: 10.1371/journal.pdig.0001022 (PMC12463258; doi:10.1371/journal.pdig.0001022)
Supplement: S1 File — 1. Figure 1: SHAP values bar graph for Feature 1 and Feature 2. 2. Synthetic Dataset 1. 3. Synthetic Dataset 2. 4. Synthetic Dataset 3. 5. Synthetic Dataset 4. 6. Synthetic Dataset 5. 7. Early Diabetes Preprocessing. 8. Heart Failure Preprocessing. (PDF) [file pdig.0001022.s001.pdf]

Individualized Machine-learning-based Clinical Assessment Recommendation System - Supplementary Documents

Devin Setiawan[1], Yumiko Wiranto[2], Jeffrey M. Girard[2], Amber Watts[2], Arian Ashourvan[2]

[1] *The University of Kansas, Department of Electrical Engineering and Computer Science, 1415 Jayhawk Blvd. Lawrence, KS 66045*

[2] *The University of Kansas, Department of Psychology, 1415 Jayhawk Blvd. Lawrence, KS 66045*

---

1. Dataset

1.1. Synthetic Dataset Generation

The basis for creating synthetic datasets 1-3 is the scenario highlighted in Supplementary Figure 1. We created a condition where two additional features display equally good predictive power as analyzed by SHAP. However, a global feature selection is shown to be insufficient as it only selects one out of the two equally good features to add for all scenarios. This limitation can lead to suboptimal inference since it overlooks the information provided by the initial feature. Synthetic datasets 1-3 are thus designed to illustrate the necessity of incorporating individualized feature recommendations, demonstrating that considering only one feature for all is inadequate for accurate inference.

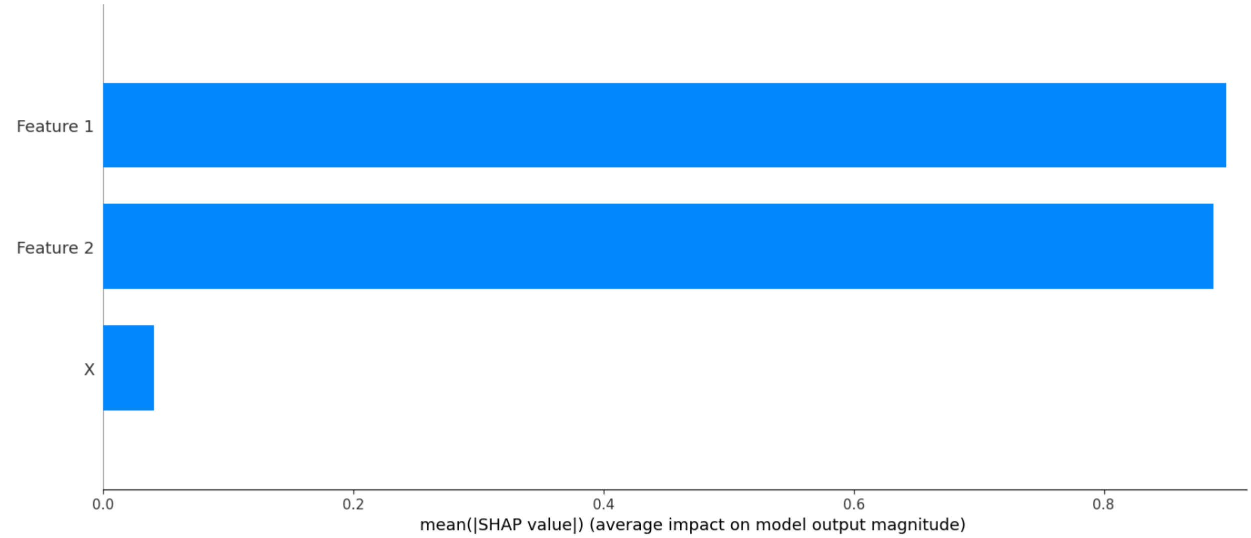

Figure 1: **SHAP values bar graph for Feature 1 and Feature 2.** The X-axis represents the SHAP value (feature importance measure) for each feature on the Y-axis. X on the Y-axis represents the initial feature set that the patient started with. The graph highlights the comparable

magnitudes for SHAP values associated with 'Feature 1' and 'Feature 2,' emphasizing the challenge faced by global feature selection methods. Traditional approaches will fall short and pick either Feature 1 or Feature 2 to add to X without any rigorous selection criteria (i.e., random selection). However, a personalized machine learning framework can adapt to individual observation and add features according to the information in X.

We created synthetic dataset 1 to have two equally good features. However, the predictiveness of each feature operates on different patient groups. Feature 1 is set to be a good predictor for X below 0.5, and Feature 2 is set to be a good predictor for X above 0.5. The variable X is the initial feature value.

Synthetic Dataset 1

Initialize *X, y, feature_1, feature_2*
X ← random values ranging from 0 to 1
y ← random values either 0 or 1
Generate Feature 1:
for all *x_i, y_i* ∈ *X, y*
  if *x_i* > 0.5 **then** *feature_1[i]* ← random values ranging from 0 to 1
  else
    if *y_i* = 0 **then** *feature_1[i]* ← random values ranging from 0 to 0.5
    if *y_i* = 1 **then** *feature_1[i]* ← random values ranging from 0.5 to 1
Generate Feature 2:
for all *x_i, y_i* ∈ *X, y*
  if *x_i* < 0.5 **then** *feature_2[i]* ← random values ranging from 0 to 1
  else
    if *y_i* = 0 **then** *feature_2[i]* ← random values ranging from 0 to 0.5
    if *y_i* = 1 **then** *feature_2[i]* ← random values ranging from 0.5 to 1

For synthetic dataset 2, Feature 1 is set to be a good predictor for X below 0.5, and Feature 2 is set to be a good predictor for X above 0.5, similar to synthetic dataset 1. However, the decision space is non-linear. For example, for X < 0.25, Feature 1 can be used to decide the outcome where Feature 1 < 0.5 means outcome = 0 and Feature 2 > 0.5 means outcome = 1. However, this decision boundary is flipped for 0.25 < X < 0.5 where Feature 1 < 0.5 means outcome = 1 and Feature 2 > 0.5 means outcome = 0.

Synthetic Dataset 2

Initialize *X, y, feature_1, feature_2*
X ← random values ranging from 0 to 1

```
    y ← random values either 0 or 1
    Generate Feature 1:
    for all x_i, y_i ∈ X, y
        if x_i > 0.5 then feature_1[i] ← random values ranging from 0 to 1
        else if x_i < 0.25
            if y_i = 0 then feature_1[i] ← random values ranging from 0 to 0.5
            if y_i = 1 then feature_1[i] ← random values ranging from 0.5 to 1
        else if x_i < 0.5
            if y_i = 1 then feature_1[i] ← random values ranging from 0 to 0.5
            if y_i = 0 then feature_1[i] ← random values ranging from 0.5 to 1
    Generate Feature 2:
    for all x_i, y_i ∈ X, y
        if x_i < 0.5 then feature_2[i] ← random values ranging from 0 to 1
        else if x_i > 0.75
            if y_i = 0 then feature_2[i] ← random values ranging from 0 to 0.5
            if y_i = 1 then feature_2[i] ← random values ranging from 0.5 to 1
        else if x_i > 0.5
            if y_i = 1 then feature_2[i] ← random values ranging from 0 to 0.5
            if y_i = 0 then feature_2[i] ← random values ranging from 0.5 to 1
```

For synthetic dataset 3, Feature 1 is set to be a good predictor for X below 0.7, and Feature 2 is set to be a good predictor for X above 0.3. This creates an overlapping predictive region where any additional feature is a good addition.

Synthetic Dataset 3

```
    Initialize X, y, feature_1, feature_2
    X ← random values ranging from 0 to 1
    y ← random values either 0 or 1
    Generate Feature 1:
    for all x_i, y_i ∈ X, y
        if x_i > 0.7 then feature_1[i] ← random values ranging from 0 to 1
        else
            if y_i = 0 then feature_1[i] ← random values ranging from 0 to 0.5
            if y_i = 1 then feature_1[i] ← random values ranging from 0.5 to 1
    Generate Feature 2:
    for all x_i, y_i ∈ X, y
        if x_i < 0.3 then feature_2[i] ← random values ranging from 0 to 1
        else
            if y_i = 0 then feature_2[i] ← random values ranging from 0 to 0.5
```

```
        if y_i = 1 then feature_2[i] ← random values ranging from 0.5 to 1
```

For synthetic dataset 4, Feature 1 is set to be a good predictor for all X values, and Feature 2 is set to be a good predictor for all X values. This creates a condition where personalization is unnecessary, as either feature can be globally selected and still produce a good prediction.

Synthetic Dataset 4

```
Initialize X, y, feature_1, feature_2
X ← random values ranging from 0 to 1
y ← random values either 0 or 1
Generate Feature 1:
for all x_i, y_i ∈ X, y
    if y_i = 0 then feature_1[i] ← random values ranging from 0 to 0.5
    if y_i = 1 then feature_1[i] ← random values ranging from 0.5 to 1
Generate Feature 2:
for all x_i, y_i ∈ X, y
    if y_i = 0 then feature_2[i] ← random values ranging from 0 to 0.5
    if y_i = 1 then feature_2[i] ← random values ranging from 0.5 to 1
```

For synthetic dataset 5, Feature 1 is set to be a good predictor for all X values while Feature 2 and 3 are set to be bad predictors for all X values. This creates a condition where personalization is not necessary, as a global feature selection will pinpoint Feature 1's usefulness and use it for all samples.

Synthetic Dataset 5

```
Initialize X, y, feature_1, feature_2, feature_3
X ← random values ranging from 0 to 1
y ← random values either 0 or 1
Generate Feature 1:
for all x_i, y_i ∈ X, y
    if y_i = 0 then feature_1[i] ← random values ranging from 0 to 0.5
    if y_i = 1 then feature_1[i] ← random values ranging from 0.5 to 1
Generate Feature 2:
for all x_i, y_i ∈ X, y
    feature_2[i] ← random values ranging from 0 to 1
Generate Feature 3:
for all x_i, y_i ∈ X, y
    feature_3[i] ← random values ranging from 0 to 1
```

1.2. Real-World Dataset Preparation

To ensure relevance and manage computational complexity, datasets were selected based on the following criteria: binary classification problem type, containing 10 to 100 features, and comprising 100 to 1000 samples. The early diabetes dataset comprises 16 features and 520 samples, while the heart failure dataset comprises 11 features and 299 samples. For the early diabetes dataset, all of the features are binary values except age. Therefore, we applied normalization to the ‘age’ feature so that its values range from 0 to 1. For the heart failure dataset, we normalize all features as they are continuous. We removed some entries with missing values. We also removed the ‘time’ column as it was unnecessary for inference.

Early Diabetes Preprocessing

```
Initialize df
df ← Read csv file 'diabetes_data_upload.csv'
Normalize Age Feature:
for all age ∈ df[‘Age’]
        do min-max normalization on age
update age values in df
save df to 'early_diabetes_normalized.csv'
```

Heart Failure Preprocessing

```
Initialize df
df ← fetch file from ‘ucirepo(id=519)’
Normalize All Feature:
for all feature ∈ df
        do min-max normalization on feature
update values in df
Remove Missing Values:
for all entries ∈ df
        if entries have missing values then
                delete entries
Remove ‘Time’ Column:
delete df[‘time’]
save df to 'heartfailure_normalized.csv'
```
