# Supplementary material for: Individualized machine-learning-based clinical assessment recommendation system
Source: PLOS Digit Health. 2025 Sep 25;4(9):e0001022. doi: 10.1371/journal.pdig.0001022 (PMC12463258; doi:10.1371/journal.pdig.0001022)
Supplement: Attachment — Submitted filename: comments.pdf [file pdig.0001022.s002.pdf]

Thank you for sharing your research. Your work on iCARE is fascinating and contributes significantly to the field of personalized clinical assessments. I appreciate the effort and innovation behind your approach and look forward to your response and any further insights you can provide.

The paper presents a novel framework, iCARE, for personalized feature selection in clinical assessments. The use of locally weighted logistic regression and SHAP value analysis is innovative and well-justified. The evaluation on synthetic and real-world datasets demonstrates the framework's potential to improve diagnostic accuracy in certain scenarios. However, there are areas where clarity, consistency, and technical accuracy can be improved. Most of the datasets used in your study are publicly available from other papers. Why did you not use real-world hospital data for validation? Would testing iCARE on clinical datasets from a healthcare institution provide stronger evidence of its practical applicability?

I could not find a GitHub repository or code for iCARE. Without access to the implementation, how can the reported high accuracy be independently verified? Could you provide additional validation, such as cross-validation results or external benchmarking, to support your claims?

1. **Individualized Feature Selection**:
   - How does the iCARE framework determine which features are most relevant for individual patients?
   - What are the advantages of using locally weighted logistic regression over traditional logistic regression in this context?
2. **SHAP Value Analysis**:
   - How does SHAP value analysis contribute to the interpretability of the iCARE framework?
   - Can you explain how SHAP values are calculated and integrated into the feature selection process?
   -

3. **Comparison with Global Approaches**:
   - What are the key differences between the iCARE framework and the Global approach in terms of feature selection and model performance?
   - Why might a Global approach fail to perform well in scenarios where individualized feature selection is critical?

4. **Synthetic Datasets**:
   - What characteristics were embedded in synthetic datasets 1-3 to make them suitable for evaluating individualized feature selection?
   - Why did iCARE show no significant advantage in synthetic datasets 4-5 and the heart failure dataset?
5. **Real-World Datasets**:
   - What specific challenges did the early-stage diabetes and heart failure datasets present for the iCARE framework?
   - How were the datasets preprocessed to ensure compatibility with the iCARE framework?
6. **Performance Metrics**:
   - Why were accuracy and AUC chosen as the primary metrics for evaluating the iCARE framework?
   - Are there any limitations to using these metrics in the context of individualized clinical assessments?

7. **Predictive Accuracy Improvements**:
   - What factors contributed to the significant improvements in accuracy and AUC for iCARE in synthetic dataset 1 and the early diabetes dataset?
   - How do the improvements in accuracy (1·5-3·5%) translate to real-world clinical decision-making?
8. **Scenarios Where iCARE Excels**:
   - In what types of clinical scenarios would iCARE be most beneficial, and why?

- Are there specific patient populations or conditions where individualized feature selection is particularly advantageous?

9. **Limitations of iCARE**:
   - What are the potential limitations of the iCARE framework, especially in datasets where features lack discernible predictive distinctions?
   - How might these limitations be addressed in future iterations of the framework?

10. **Personalized Medicine**:
    - How does iCARE contribute to the broader field of personalized medicine?
    - What are the ethical considerations when implementing machine learning frameworks like iCARE in clinical practice?
11. **Integration into Healthcare Systems**:
    - What steps would be necessary to integrate iCARE into existing healthcare systems and workflows?
    - How might clinicians and healthcare providers be trained to use and interpret the results of iCARE effectively?
12. **Cost and Resource Implications**:
    - What are the potential costs and resource requirements associated with implementing iCARE in a clinical setting?
    - How might the framework be adapted for use in resource-constrained environments?

13. **Scalability and Generalizability**:
    - How scalable is the iCARE framework to other medical conditions beyond diabetes and heart failure?
    - What modifications would be needed to apply iCARE to non-clinical datasets or other domains?
14. **Advanced Machine Learning Techniques**:
    - Could deep learning or other advanced machine learning techniques be integrated into the iCARE framework to further enhance its performance?

- What are the trade-offs between using simpler models like logistic regression and more complex models in this context?

15. **Long-Term Impact**:
    - What long-term impact could iCARE have on the field of clinical diagnostics and patient outcomes?
    - How might the framework evolve as more data becomes available and machine learning techniques advance?

I. **Methodology and Framework**:
   - The iCARE framework uses locally weighted logistic regression and SHAP value analysis for personalized feature selection. How does the locally weighted logistic regression differ from traditional logistic regression, and why is it more suitable for individualized clinical assessments?
   - Could you explain how the sample weight calculation module ensures that the model focuses on patients with similar characteristics to the incoming patient?

II. **SHAP Value Analysis**:
   - SHAP values are used to quantify feature importance in the iCARE framework. How do SHAP values contribute to the interpretability of the model, and why are they particularly useful in clinical decision-making?
   - Are there any limitations to using SHAP values in this context, especially when dealing with highly imbalanced datasets or non-linear relationships?

III. **Synthetic Datasets**:
   - The paper uses synthetic datasets 1-5 to evaluate the iCARE framework. What specific characteristics were embedded in synthetic datasets 1-3 to simulate ideal scenarios, and how do these characteristics validate the framework's ability to provide personalized recommendations?
   - In synthetic datasets 4-5, where iCARE showed no significant advantage, what does this imply about the limitations of personalized feature selection in certain clinical scenarios?

IV. **Real-World Datasets**:

- The iCARE framework was tested on real-world datasets, including early-stage diabetes and heart failure datasets. Why did iCARE show significant improvements in the early diabetes dataset but not in the heart failure dataset?
- What does this discrepancy suggest about the types of clinical conditions or datasets where personalized feature selection is most beneficial?

V. **Comparison with Global Approaches**:
   - The paper compares iCARE with a Global approach. What are the key differences in performance between these two approaches, and under what conditions does iCARE outperform the Global approach?
   - Are there scenarios where a Global approach might still be preferable over iCARE, and why?

VI. **Sample Weighting**:
   - Sample weighting is a critical component of the iCARE framework. How does the weighting strategy ensure that the model prioritizes patients with similar characteristics, and what are the potential challenges in implementing this strategy in real-world clinical settings?
   - Could you discuss how sample weighting might be adapted for use with other machine learning models beyond logistic regression?

VII. **Limitations of iCARE**:
   - The paper mentions several limitations of the iCARE framework, including the lack of an automatic mechanism to determine whether a dataset warrants personalized feature recommendation. How might this limitation impact the scalability and practicality of iCARE in clinical settings?
   - What future research directions could address this limitation and improve the framework's applicability?

VIII. **Comparison with Other Frameworks**:
   - The paper compares iCARE with the imputation-based explanation-guided (Eguided) feature selection method. What are the key differences in performance between these two frameworks, and why does iCARE generally outperform Eguided in the tested datasets?

- How might the choice of machine learning model (e.g., logistic regression vs. XGBoost) influence the relative performance of these frameworks?

IX. **Clinical Implications**:

- The iCARE framework aims to enhance diagnostic accuracy through personalized feature selection. What are the potential clinical implications of this framework, particularly in terms of improving patient outcomes and reducing healthcare costs?
- How might clinicians and healthcare providers be trained to effectively use and interpret the results of iCARE in their practice?

X. **Future Directions**:

- The paper suggests that future work could focus on developing robust criteria to assess the need for personalization more efficiently. What specific criteria or indicators might be useful in determining whether a dataset is suitable for personalized feature selection?
- How might advancements in machine learning, such as deep learning or reinforcement learning, be integrated into the iCARE framework to further enhance its capabilities?

Corrections and Suggestions:

1. **Decimal Notation**:
    - Replace hyphens (-) and middle dots (·) with periods (.) for decimal notation. For example:
        - "0-999" → "0.999"
        - "1-000" → "1.000"
        - "1-5-3-5%" → "1.5-3.5%"
    - This correction applies to the abstract, results, tables, and throughout the manuscript.
2. **Consistent Capitalization**:
    - Ensure consistent use of "iCARE" (not "ICARE") throughout the paper.
3. **Table Formatting**:
    - Use standard decimal notation in tables (e.g., "0.310" instead of "0-310").
    - Ensure proper alignment and readability of tables.
4. **Clarity in Phrasing**:

- Rephrase sentences for better clarity. For example:
    - Original: "iCARE will excel in scenarios where patients with similar profiles benefit from similar recommendations."
    - Revised: "iCARE performs best in scenarios where patients with similar profiles benefit from similar recommendations."

5. **Methodology Explanation**:
    - Provide more detailed explanations or visual aids (e.g., flowcharts) for the sample weight calculation module and the overall workflow of the iCARE framework.
6. **Standardize Terminology**:
    - Ensure consistent use of terms like "iCARE," "SHAP," and "locally weighted logistic regression" throughout the paper.

Conclusion:

The iCARE framework is a promising approach to personalized feature selection in clinical assessments. Addressing the questions and corrections outlined above will strengthen the manuscript and enhance its clarity, consistency, and impact. I recommend that the authors carefully review these points and revise the paper accordingly.
