# Supplementary material for: Individualized machine-learning-based clinical assessment recommendation system
Source: PLOS Digit Health. 2025 Sep 25;4(9):e0001022. doi: 10.1371/journal.pdig.0001022 (PMC12463258; doi:10.1371/journal.pdig.0001022)
Supplement: Attachment — Submitted filename: Response to Reviewer.pdf [file pdig.0001022.s004.pdf]

Response to Reviewer

Reviewer #1

Dear Authors,

Thank you for your submission. Your work on iCARE presents an innovative approach to personalized clinical assessment. I appreciate the effort in integrating locally weighted models and SHAP analysis to improve diagnostic accuracy.

However, I have a few concerns that I believe would strengthen the study:

Generalizability & Validation – Have you tested iCARE on real-world hospital datasets rather than relying solely on synthetic and publicly available data? This would enhance its clinical applicability.

Thank you for your thoughtful suggestion regarding the validation of iCARE on real-world hospital datasets. We agree that testing on clinical data from hospitals would significantly enhance the applicability of our approach. However, due to restrictions on accessing real-world patient data, we are currently limited to using publicly available datasets. We provide the following text in the discussion section titled "*Limitations and Future Directions*" to highlight the importance of validation on real-world datasets:

"Additionally, while iCARE was evaluated on medically relevant and clinically representative datasets, these datasets do not fully substitute for real-world hospital data. To ensure the relevance of our study, we carefully selected datasets that approximate clinical scenarios; however, further validation on real-world hospital data remains critical. We hope to explore potential collaborations to facilitate this aspect of future research and strengthen iCARE's applicability in clinical practice." (p23)

Performance Benchmarking – How does iCARE compare to existing feature selection methods such as LASSO or SFS? Have you conducted ablation studies or statistical significance tests to confirm its advantages?

We appreciate your suggestion regarding the comparison of iCARE with existing feature selection methods. In response to your feedback, we have updated Figure 12 (response document Figure 1) and added Section 3.4 (Comparison with Other Frameworks) to include results using LASSO and Sequential Feature Selection (SFS) in addition to the previously included Global SHAP method and Explanation-Guided method. This addition provides a more comprehensive performance benchmarking of iCARE against established feature selection techniques. We hope

this update addresses your concern and further strengthens the evaluation of our approach. We provided the following text in the Results section titled "*Comparison with Other Frameworks*" to report these new findings:

"We also provide comparisons with other global feature selection methods which are SHAP based (Global), SFS, and Lasso. As shown in Figure 12, iCARE achieved a +6% higher ROC-AUC score on average in the Early Diabetes Dataset and +12.1% higher ROC-AUC score on average in the Heart Disease Dataset over Eguided. Over the global feature selection methods, iCARE showed around the same increase in performance." (p18)

and the following text to the figure titled "Figure 12: Performance Comparison of Feature Selection Methods Across Three Datasets." to adjust for the new methods that were added:

"Figure 12: Performance Comparison of Feature Selection Methods Across Three Datasets. This figure presents a comparative evaluation of AUC-ROC and Accuracy across different feature selection approaches on three real-world datasets: (a) Early Diabetes, (b) Heart Failure, and (c) Heart Disease. The graphs illustrate the performance of five feature selection methods which are Global (blue), SLS (orange), LASSO (green), iCARE (red), and eGuided (purple), across varying feature subsets. The x-axis represents the number of selected features, while the y-axis shows the corresponding AUC-ROC and Accuracy scores, providing insights into the effectiveness of each method in optimizing predictive performance." (p20)

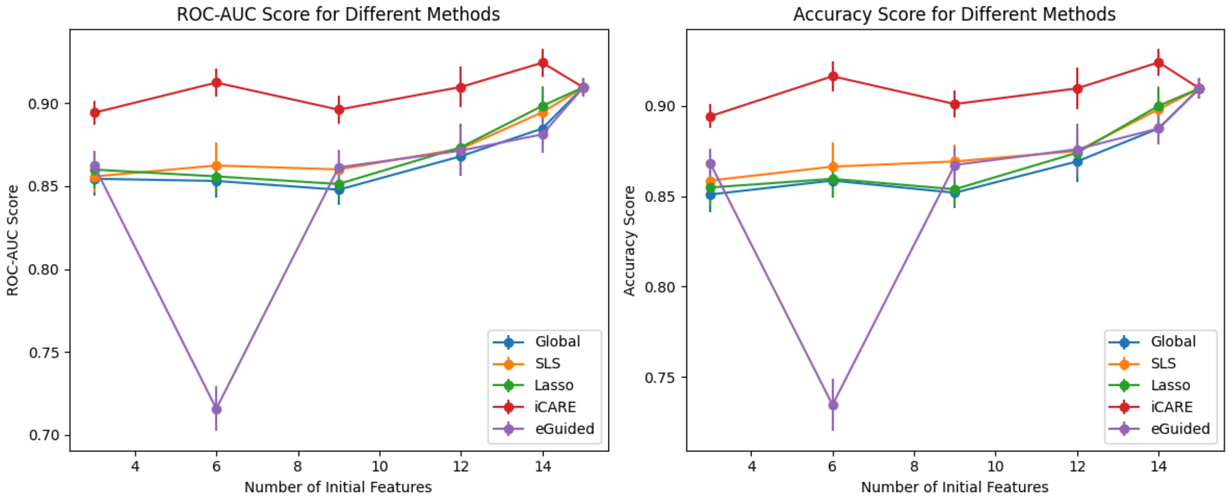

(a) Early Diabetes Dataset

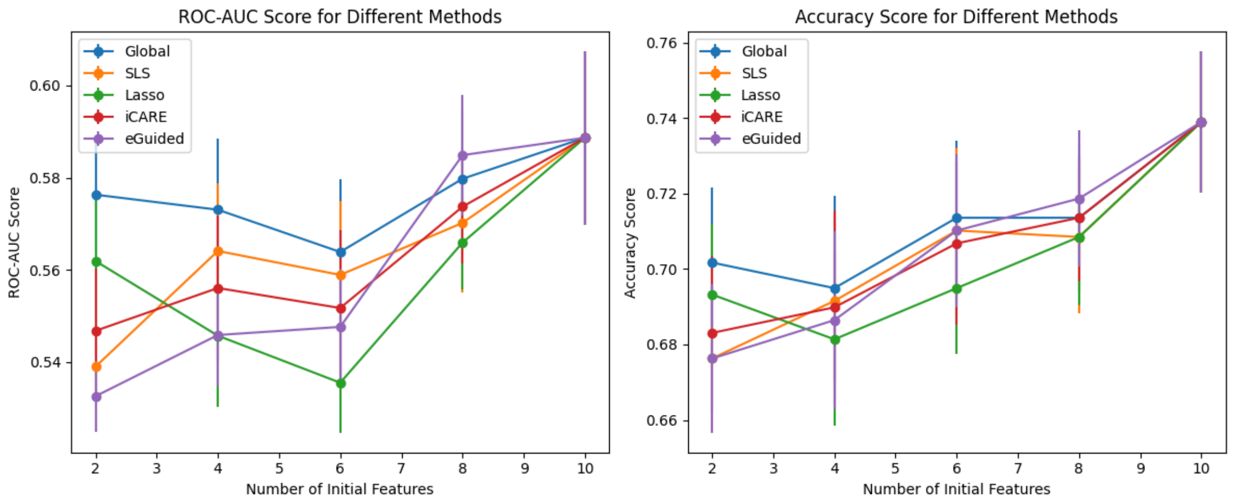

(b) Heart Failure Dataset

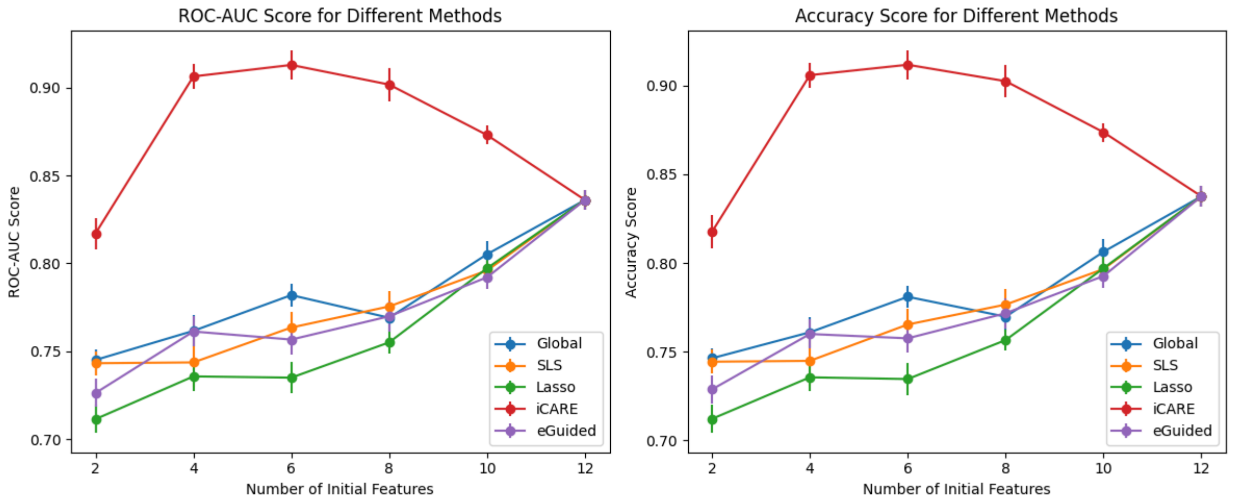

(c) Heart Disease Dataset

Figure 1: Performance Comparison of Feature Selection Methods Across Three Datasets.

Reproducibility – I couldn't find any reference to a GitHub repository or code availability. Without access to the implementation, how can the results be independently verified?

Thank you for your feedback on code availability. We have now added a Code Availability section after the conclusion to clearly outline the availability of our implementation. In addition, the GitHub repository now includes all the scripts necessary for the complete reproduction of our results and manuscript figures. We provided the following text under "Code Availability":

"The implementation of the iCARE framework and also the datasets used in this study can be found in the following github link: https://github.com/DevinRS/iCARE. Code is written in Python 3.11.9." (p24)

Clinical & Ethical Considerations – How does iCARE ensure fairness in feature selection across different patient demographics? Are privacy concerns (e.g., HIPAA, GDPR) addressed in the framework?

Thank you for raising these important points regarding fairness and privacy. While iCARE does not explicitly incorporate fairness constraints, its feature selection process is based on locally weighted learners, which naturally accounts for patient similarity. By calculating similarity scores between an incoming patient and all available patients in the dataset, we can assess whether the incoming patient has sufficiently similar counterparts—including demographic factors such as race. This allows us to preemptively evaluate the reliability of the decision-making process. If an incoming patient has no sufficiently similar cases in the dataset, we can flag them as an outlier and advise caution in using iCARE's recommendations. This built-in assessment helps mitigate potential biases by ensuring that predictions are only made when meaningful comparisons exist. To highlight this very critical point raised by the reviewer, we added the following text in the Discussion section titled "*Limitations and Future Directions*":

"Lastly, Ensuring fairness in feature selection is crucial, particularly when population representation is imbalanced. iCARE does not explicitly enforce fairness constraints, but its use of locally weighted learners inherently accounts for patient similarity, including demographic factors like race. By computing similarity scores, iCARE can identify whether an incoming patient has sufficiently comparable cases in the dataset. If not, the system can flag them as outliers, prompting caution in interpreting recommendations. Future work could build on this feature to formally incorporate fairness-aware methodologies and improve iCARE's reliability across diverse populations." (p24)

Regarding privacy, while iCARE operates on publicly available datasets, future implementations will consider compliance with regulations such as HIPAA and GDPR should real-world patient data be incorporated. In the Discussion section titled 'Limitations and Future Directions,' we addressed a critical point concerning the security risks associated with the datasets used for model training, as well as the potential vulnerability of models trained on sensitive private data to misuse or abuse:.

"Regarding privacy, future implementations will ensure to account for compliance with regulations such as HIPAA and GDPR when incorporating real-world patient data. Furthermore, it is important to acknowledge the security risks associated with the datasets used for model training. Models trained on sensitive private data can be vulnerable to misuse or abuse,

particularly in scenarios where adversaries may attempt to extract identifiable information or exploit model behavior. Future work should also consider integrating privacy-preserving techniques such as differential privacy or secure multi-party computation to protect both the data and the individuals represented in it." (p23)

Overall, this is a promising study, but further clarification on validation, benchmarking, and reproducibility would enhance its impact. I look forward to your response and revisions.

Best regards,

<u>Reviewer #2:</u>

1. Describe dataset features in more details and its total size and size of (train/test) as a table and make it public.

Thank you for your feedback on the datasets information. We have now provided a more detailed explanation regarding the features available in each dataset, as well as the train/test split as a table in the manuscript. We have added the following text in the Methods section titled "*Dataset*":

"The early-stage diabetes dataset contains 16 features such as age, gender, polyuria, polydipsia, sudden weight loss, weakness, and other symptoms commonly associated with diabetes, along with a binary class label. The heart failure dataset includes 13 clinical attributes, including age, anaemia, serum creatinine, ejection fraction, and a binary death event label. The heart disease dataset comprises 14 features such as age, sex, chest pain type, resting blood pressure, cholesterol, fasting blood sugar, and other cardiovascular indicators, with a binary target label indicating the presence or absence of heart disease." (p7-8)

We also added Table 1 (response document Table 1) to provide information on the size of each dataset splitting and provided the following caption:

"The table shows the summary of dataset sizes and their train/test splits. The split follows a random 80/20 train/test split per iteration. Each dataset is listed with the number of subjects used for training, testing, and the total number of subjects." (p8)

Dataset	Training Set	Testing Set	Total
Early Diabetes	416	104	520

Heart Failure	239	60	299
Heart Disease	820	205	1025

Table 1. Dataset Size and Splitting

2. Pseudocode and algorithm steps need to be inserted.

Thank you for highlighting this important point. We have now provided a pseudocode of the iCARE framework in addition to the existing architecture figure to provide clarity for the reader. We added Algorithm 1 and 2 (response document Algorithm 1 and Algorithm 2), and also added to Section 2.1 (*Framework Architecture*) an explanation in terms of the pseudocode. The following text is added to the Methods section under "*Framework Architecture*":

"Figure 1 illustrates the overall architecture of the iCARE framework. To complement this high-level view, Algorithms 1 and 2 provide a detailed step-by-step pseudocode representation of the framework's two core processes: calculate weight and generate iCARE feature recommendation, respectively. These algorithms formalize the logic described above and offer clarity on how the framework operates on incoming patient data." (p5)

We provide the following caption for algorithm 1:

"This algorithm calculates the sample weights for personalized modeling. Given a single patient case and a labeled dataset, this algorithm computes the Euclidean distance between the incoming patient and all known cases. The inverse of these distances is then used to assign weights, ensuring that more similar cases exert greater influence during model training." (p5)

And the following caption for Algorithm 2:

"This algorithm computes patient-specific weights and uses them to train a personalized logistic regression model. SHAP is then applied to the trained model to quantify feature importance. The features are ranked based on their contribution to the model's prediction, allowing the framework to recommend the most informative features for improving predictive accuracy." (p6)

3. Time spent need to be measured in the experimental results.

We appreciate your suggestions on including a measure of time for running the experiment. In response to your feedback we have added a new section to our "*Findings and Interpretation*" titled "*Timing Analysis*":

“To evaluate the practicality of each feature recommendation approach in real-world scenarios, we conducted a timing analysis using two different datasets: the Early Diabetes Dataset and a synthetic dataset created for controlled experimentation. The primary objective is to assess how the number of patients requiring recommendations and the number of available features affect the computational efficiency of the methods. Importantly, our experimental setup simulates a real-world clinical setting, where patients arrive one at a time and recommendations are made on a per-patient basis. This avoids assumptions of batch processing or the possibility of precomputing feature recommendations. This setup is more realistic because in actual deployments, new patients may update the pool of known cases, invalidating any precomputed global recommendations.

The first part of the experiment investigates how the number of patients requiring feature recommendation affects the runtime. Using the Early Diabetes Dataset, we simulated a scenario where each patient starts with three known features—‘Age’, ‘Gender’, and ‘Obesity’—and the remaining features are to be recommended. We varied the number of patients (N = 20, 40, 60, 80, 100), ensuring that each recommendation is performed sequentially, without batching. We plotted the result in Figure 13, the left graph, to show the timing relationship. In this setting, we observed that the Global SHAP method consistently yielded the fastest recommendation times. iCARE, Sequential Forward Selection (SFS) and LASSO required more time, and eGuided was the slowest among the methods tested.

The second part of the experiment focused on how the number of available features for recommendation influences timing. To isolate this variable, we constructed a synthetic dataset consisting of 500 samples and 100 randomly generated features, with an added binary class label. We then varied the number of features available for selection (N = 1, 20, 40, 60), again applying each recommendation method to one patient at a time. As seen in Figure 13, the right graph, Global SHAP and iCARE outperform the other methods. Their runtimes remained relatively stable despite the increasing number of features. eGuided and SFS showed moderate performance with a linear trend, LASSO demonstrated significantly higher runtimes.

These findings align well with the computational structure of each method. Global SHAP is the most efficient due to its lightweight process of training a logistic regression model and calculating SHAP values, which are fast and insensitive to both sample size and feature dimensionality. iCARE leverages SHAP for personalized recommendations, making it similarly insensitive to feature count, though it incurs additional linear time with the number of patients due to weight calculations for each sample. SFS is slower because it iteratively adds one feature at a time based on model performance, requiring multiple rounds of model training. LASSO also requires retraining and regularization over the feature space, making it computationally expensive. Lastly, eGuided is the most computationally intensive because it identifies the 100

most similar cases, imputes unknown features to generate synthetic samples, and then computes SHAP values on those samples.” (p20-21)

We also added an accompanying Figure 13 (response document Figure 2) with the following caption:

“Figure 13: Timing Performance of Feature Recommendation Methods Across Varying Sample Sizes and Feature Dimensions. Runtime comparisons for five feature recommendation methods: Global SHAP, SFS, LASSO, iCARE, and eGuided, under two experimental conditions. The left panel shows how the runtime scales with the number of patients (N = 20 to 100) using the Early Diabetes Dataset, where each patient begins with three known features. The right panel displays runtime behavior as the number of available features increases (1 to 60) using a synthetic dataset of 500 samples and 100 total features.” (p21-22)

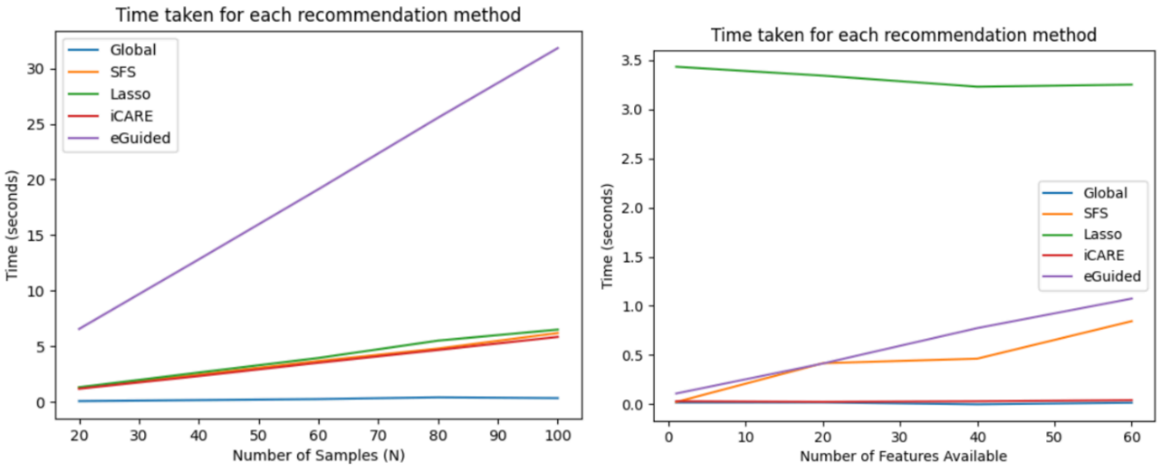

Figure 2: Timing Performance of Feature Recommendation Methods Across Varying Sample Sizes and Feature Dimensions.

4. Limitation and Discussion Sections need to be inserted.

Thank you for your thoughtful feedback. We would like to kindly point out that our manuscript includes a Discussion section, titled “*4. Discussion,*” which contains a dedicated subsection, “*4.4 Limitations and Future Directions.*” However, we fully acknowledge that the original limitations section may have lacked sufficient depth in certain areas. In response to this and in alignment with the suggestions from other reviewers, we have revised and expanded this subsection to provide a more thorough discussion. Specifically, we have addressed the limitation of not utilizing real-world hospital data:

“Additionally, while iCARE was evaluated on medically relevant and clinically representative datasets, these datasets do not fully substitute for real-world hospital data. To ensure the

relevance of our study, we carefully selected datasets that approximate clinical scenarios; however, further validation on real-world hospital data remains critical. We hope to explore potential collaborations to facilitate this aspect of future research and strengthen iCARE's applicability in clinical practice." (p23)

We also addressed potential privacy and security concerns related to our framework:

"Regarding privacy, future implementations will be ensured to account for compliance with regulations such as HIPAA and GDPR when incorporating real-world patient data. Furthermore, it is important to acknowledge the security risks associated with the datasets used for model training. Models trained on sensitive private data can be vulnerable to misuse or abuse, particularly in scenarios where adversaries may attempt to extract identifiable information or exploit model behavior. Future work should also consider integrating privacy-preserving techniques such as differential privacy or secure multi-party computation to protect both the data and the individuals represented in it." (p23)

"Lastly, Ensuring fairness in feature selection is crucial, particularly when population representation is imbalanced. iCARE does not explicitly enforce fairness constraints, but its use of locally weighted learners inherently accounts for patient similarity, including demographic factors like race. By computing similarity scores, iCARE can identify whether an incoming patient has sufficiently comparable cases in the dataset. If not, the system can flag them as outliers, prompting caution in interpreting recommendations. Future work could build on this feature to formally incorporate fairness-aware methodologies and improve iCARE's reliability across diverse populations." (p24)

We have also retained and clarified our previously stated limitations regarding the model itself. We hope these improvements strengthen the manuscript and adequately address your concerns.

5. All metrics need to be calculated in the experimental results.

Thank you very much for pointing this out. We appreciate your careful attention to the consistency of our experimental results. We have revised Figure 12 (shown as Figure 1 in the response document) to include both Accuracy and ROC-AUC, ensuring that all relevant metrics are presented consistently throughout the manuscript. We hope this correction improves the clarity and completeness of our results.

6. The parameters used for the analysis must be provided in table

Thank you for your insightful suggestion. In our study, we employed logistic regression with default parameters from the scikit-learn library for all feature selection methods. To address this,

we stated explicitly our selection of parameters to enhance the clarity of our methodology. We have added the following sentence to the Methods section under "*Framework Architecture*":

"Using these weights, a weighted logistic regression (default params: penalty='l2', solver='lbfgs', c='1') is trained using the pool of known cases." (p3)

7. The architecture of the proposed model must be provided

Thank you for your thoughtful comment. The architecture of our proposed model is presented in Figure 1 of the original manuscript, where we highlight the five key modules comprising the iCARE framework. To improve clarity and in response to your helpful suggestions regarding the inclusion of pseudocode, we have now added a pseudocode representation of the architecture (see response document, Algorithms 1 and 2). The following text is also added to the Methods section under "*Framework Architecture*":

"Figure 1 illustrates the overall architecture of the iCARE framework. To complement this high-level view, Algorithms 1 and 2 provide a detailed, step-by-step pseudocode representation of the framework's two core processes: calculating weight and generating iCARE feature recommendations, respectively. These algorithms formalize the logic described above and offer clarity on how the framework operates on incoming patient data." (p5)

We provide the following caption for Algorithm 1:

"This algorithm calculates the sample weights for personalized modeling. Given a single patient case and a labeled dataset, this algorithm computes the Euclidean distance between the incoming patient and all known cases. The inverse of these distances is then used to assign weights, ensuring that more similar cases exert greater influence during model training." (p5)

And the following caption for Algorithm 2:

"This algorithm computes patient-specific weights and uses them to train a personalized logistic regression model. SHAP is then applied to the trained model to quantify feature importance. The features are ranked based on their contribution to the model's prediction, allowing the framework to recommend the most informative features for improving predictive accuracy." (p6)

8. Comparison with similar studies on a similar dataset need to be inserted (with references).

We appreciate your suggestion and acknowledge that we did not include relevant studies that utilize the same dataset. We have included relevant studies that used the same dataset, as suggested. Under the Methods section titled "*Dataset,*" we have added the following statement:

"These datasets have been used for evaluating diagnosis using various machine learning approaches and in developing identification systems in healthcare." (p8)

With the following citations:

30 Islam MdR, Banik S, Rahman KN, Rahman MM. A comparative approach to alleviating the prevalence of diabetes mellitus using machine learning. Computer Methods and Programs in Biomedicine Update 2023; 4: 100113.

31 Souza VS, A. Lima D. Identifying Risk Factors for Heart Failure: A Case Study Employing Data Mining Algorithms. JDSIS 2023; 2: 161–73.

32 R. S, P. K. Heart disease severity level identification system on Hyperledger consortium network. PeerJ Computer Science 2023; 9: e1626.

9. The cost associated with deploying these deep learning models, including the necessary hardware and software, is not addressed.

Thank you for this helpful comment. We would like to clarify that the iCARE framework is model-agnostic, meaning it can be used with any predictive modeling approach, including but not limited to deep learning models, support vector machines (SVMs), decision trees, and logistic regression. In our experiments, we chose logistic regression due to its interpretability and computational efficiency, as our goal was to showcase the utility of iCARE in individualized feature selection rather than to benchmark specific model architectures. As such, estimating deployment costs for deep learning models would be beyond the scope of this work, since we do not rely on deep learning in our implementation. Therefore, to increase clarity for readers, we have added a new subsection under the Methods section (Section 2.5: Implementation Details) to describe the hardware and software setup used in our study and to highlight the generalizability and low-cost nature of our framework:

"The iCARE framework is designed to be model-agnostic and can be used in conjunction with any machine learning algorithm suitable for the problem at hand. While this study employs logistic regression to demonstrate the framework's capacity for individualized feature selection and interpretability, it can be integrated with more complex models such as deep neural networks, random forests, or support vector machines, depending on the user's needs. All experiments in this study were implemented in Python 3.11.9 using the scikit-learn library for logistic regression and the SHAP library for feature importance estimation. These experiments

were conducted on a consumer-grade Windows laptop without specialized hardware (e.g., TPUs or high-performance computing).” (p8)

10. Address the accuracy/improvement percentages in the abstract and in the conclusion sections, as well as the significance of these results.

Thank you for your suggestion. We have added a summary of the observed improvements in predictive accuracy and AUC in both the abstract and the conclusion. This is the text we added to the abstract:

“In the early diabetes and heart disease dataset, iCARE shows improvements of 6-12% in accuracy and AUC across different numbers of initial features over other feature selection methods.” (p1)

This is the text we added to our Conclusion section:

“In our experiments on early stage diabetes and heart disease datasets, iCARE demonstrated improvements of 6-12% in both accuracy and AUC compared to traditional feature selection methods. These improvements highlight the practical benefits of personalization in enhancing prediction performance, which can contribute to more accurate clinical decisions.” (p24)

11. The authors need to make a clear proofread to avoid grammatical mistakes and typo errors.

We appreciate the reviewer’s suggestion and we have carefully proofread the manuscript and corrected all grammatical mistakes and typographical errors to improve the overall clarity and readability of the paper.

12. Add future work in last section (conclusion) (if any)

We appreciate your suggestion and have taken steps to improve our paper to include future work in the conclusion section. Here’s a passage that we added to the Conclusion section, highlighting a summary of future directions:

“Future work will focus on developing automated mechanisms to determine when personalization is warranted, optimizing the framework for real-time applications, and validating its effectiveness on real-world hospital datasets to strengthen iCARE’s robustness in diverse settings.” (p24)

13. Enhance the clarity of the Figures by improving their resolution.

We appreciate the reviewer's feedback. We have adjusted the figures to improve their resolution and ensured they meet the journal's guidelines.

14. The authors need to add recent articles in related work and update them.

Addressed below in response to point 15

15. To improve the Related Work and Introduction sections authors are recommended to review this highly related research work paper:
a) Secure and Transparent Lung and Colon Cancer Classification Using Blockchain and Microsoft Azure
b) Optimizing epileptic seizure recognition performance with feature scaling and dropout layers
c) Advances in ECG and PCG-based cardiovascular disease classification: a review of deep learning and machine learning methods
d) Feature reduction for hepatocellular carcinoma prediction using machine learning algorithms
e) The power of deep learning in simplifying feature selection for hepatocellular carcinoma: a review
f) Utilizing machine learning to analyze trunk movement patterns in women with postpartum low back pain
g) Employing machine learning for enhanced abdominal fat prediction in cavitation post-treatment
h) Machine learning insights into scapular stabilization for alleviating shoulder pain in college students
i) Revolutionizing core muscle analysis in female sexual dysfunction based on machine learning
j) Utilizing convolutional neural networks to classify monkeypox skin lesions
k) Predicting female pelvic tilt and lumbar angle using machine learning in case of urinary incontinence and sexual dysfunction
l) Hepatitis C Virus prediction based on machine learning framework: a real-world case study in Egypt

Thank you for your valuable feedback regarding the Related Work and Introduction sections. We have carefully reviewed the suggested list of highly related studies and appreciate the breadth and relevance of the recommendations. To strengthen the manuscript, we have selected and incorporated several studies from the list that align closely with our research objectives and methodology. These have been integrated into the "Introduction" section along with their citation as follows:

"Prediction of possible outcomes and prediction of treatment impact are two important components of today's medical care and customized healthcare." (p2)

Citation: 1 Abdel Hady DA, Mabrouk OM, Abd El-Hafeez T. Employing machine learning for enhanced abdominal fat prediction in cavitation post-treatment. Sci Rep 2024; 14: 11004.

"Machine learning (ML) approaches are commonly used for predicting and classifying diseases that are precisely utilized as an efficient tool for doctors and specialists." (p2)
Citation: 2 Mamdouh Farghaly H, Shams MY, Abd El-Hafeez T. Hepatitis C Virus prediction based on machine learning framework: a real-world case study in Egypt. Knowl Inf Syst 2023; 65: 2595–617.

"A specific example in oncology would be Hepatocellular carcinoma (HCC), a primary liver cancer with an aggressive nature, and that despite research, the prognosis remains unfavorable, with considerable unmet needs in providing personalized treatment options." (p2)
Citation: 6 Mostafa G, Mahmoud H, Abd El-Hafeez T, E.ElAraby M. The power of deep learning in simplifying feature selection for hepatocellular carcinoma: a review. BMC Med Inform Decis Mak 2024; 24: 287.

Reviewer #3:

Review Report

Summary of the Paper:
The manuscript presents a novel machine-learning framework, the Individualized Clinical Assessment Recommendation System (iCARE), which employs locally weighted logistic regression and SHAP (Shapley Additive Explanations) value analysis to tailor feature selection to individual patient characteristics. The authors evaluate the framework on both synthetic and real-world datasets, demonstrating its effectiveness in enhancing diagnostic accuracy, particularly in scenarios where additional features exhibit distinct predictive capabilities. The paper is well-structured, and the methodology is sound, with clear explanations of the framework's architecture and experimental design.

Strengths:
1. **Innovative Approach:** The iCARE framework addresses a critical gap in clinical assessments by providing personalized feature recommendations, which is a significant advancement over traditional global feature selection methods.

2. **Comprehensive Evaluation:** The authors thoroughly evaluate the framework using both synthetic and real-world datasets, providing robust evidence of its effectiveness.
3. **Clear Methodology:** The paper provides a detailed explanation of the framework's architecture, including the use of SHAP values for feature importance, which enhances the interpretability of the model.
4. **Practical Implications:** The framework has the potential to improve diagnostic accuracy in clinical settings, particularly in personalized medicine, where individualized approaches are crucial.

Weaknesses and Suggestions for Improvement:

1. **Lack of Discussion on Related Work:**
- The paper would benefit from a more in-depth discussion of related work, particularly in the context of explainable machine learning (ML) and personalized medicine. Specifically, the authors should cite and discuss recent studies that have applied explainable ML to clinical data, such as:
- **D'Amore et al. (2025):** This study explores explainable ML to predict and differentiate Alzheimer's progression by sex, which is highly relevant to the iCARE framework's focus on personalized feature selection. The authors should discuss how their approach compares to or could be integrated with the methods proposed by D'Amore et al.
- **Angelini et al. (2024):** This paper uses explainable ML to unravel sex differences in Parkinson's disease, providing insights into how personalized feature selection can be applied to neurodegenerative diseases. The authors should consider how their framework could be extended to address similar challenges in Parkinson's disease or other neurological conditions.

2. **Limitations of the Framework:**
- The authors acknowledge some limitations, such as the lack of an automatic mechanism to determine whether a dataset warrants personalized feature recommendation. However, they should also discuss the potential challenges of scaling the framework to larger datasets or more complex clinical scenarios. For example, the computational cost of training locally weighted models for each patient could be prohibitive in real-time clinical settings.
- Additionally, the authors should explore the potential ethical implications of using personalized feature selection, particularly in terms of data privacy and the potential for bias in the recommendations.

3. **Comparison with Other Frameworks:**
- While the authors compare iCARE with a global feature selection approach and an imputation-based explanation-guided method, they should also consider comparing their

framework with other state-of-the-art personalized feature selection methods. This would provide a more comprehensive evaluation of iCARE performance and highlight its unique contributions.

4. **Clarification on SHAP Values:**
- The authors should provide more detailed explanations of how SHAP values are calculated and interpreted within the iCARE framework. This would help readers better understand the role of SHAP in personalized feature selection and its impact on the model's recommendations.

5. **Future Directions:**
- The authors should expand on their discussion of future directions, particularly in terms of integrating iCARE with other machine learning algorithms or extending its application to other domains beyond clinical assessments. For example, they could explore how the framework could be adapted for use in predictive maintenance, financial forecasting, or other fields where personalized feature selection is valuable.

Recommendation:
The paper presents a novel and promising approach to personalized feature selection in clinical assessments, with strong potential for improving diagnostic accuracy. However, the manuscript would benefit from a more thorough discussion of related work, particularly in the context of explainable ML and personalized medicine, as well as a more detailed exploration of the framework's limitations and future directions.

Recommendation: **Accept with Major Revisions**

Specific Suggestions for Revision:
1. **Cite and Discuss Relevant Literature:**
- Add a discussion of **D'Amore et al. (2025)** and **Angelini et al. (2024)** in the introduction or related work section, highlighting how their approaches to explainable ML and personalized medicine relate to the iCARE framework.
- Discuss how the iCARE framework could be applied or extended to address challenges similar to those explored in these studies, such as predicting disease progression by sex or unraveling sex differences in neurodegenerative diseases.

Thank you for the thoughtful suggestion to include and discuss the works of D’Amore et al. (2025) and Angelini et al. (2024). We acknowledge that the paper would benefit from the

inclusion of more recent and relevant studies, as also noted by other reviewers. We have added the following statement under Methods titled "*Framework Architecture*" to emphasized the use of SHAP values in other medically related research:

"SHAP has emerged as a tool for interpreting machine learning models in medical research, providing insights into the contribution of individual features to predictions. Studies utilizing SHAP have successfully identified key clinical features for disease prediction, such as neuropsychological test scores in Alzheimer's disease and motor and non-motor symptoms in Parkinson's disease." (p4)

With the following citations:

25 Angelini G, Malvaso A, Schirripa A, et al. Unraveling sex differences in Parkinson's disease through explainable machine learning. Journal of the Neurological Sciences 2024; 462: 123091.

26 D'Amore FM, Moscatelli M, Malvaso A, et al. Explainable machine learning on clinical features to predict and differentiate Alzheimer's progression by sex: Toward a clinician-tailored web interface. Journal of the Neurological Sciences 2025; 468: 123361.

2. **Expand on Limitations and Ethical Considerations:**
- Provide a more detailed discussion of the limitations of the iCARE framework, particularly in terms of scalability and computational cost.
- Address potential ethical concerns, such as data privacy and bias in personalized feature recommendations.

We appreciate the insightful feedback regarding the limitations and ethical considerations of the iCARE framework. We acknowledge the importance of these points and, in light of this and similar concerns raised by other reviewers, we have expanded the Limitations section of the manuscript to address these issues more thoroughly. Specifically, we provide the following text in the discussion section titled "*Limitations and Future Directions*" to address the limitation of not utilizing real-world hospital data:

"Additionally, while iCARE was evaluated on medically relevant and clinically representative datasets, these datasets do not fully substitute for real-world hospital data. To ensure the relevance of our study, we carefully selected datasets that approximate clinical scenarios; however, further validation on real-world hospital data remains critical. We hope to explore potential collaborations to facilitate this aspect of future research and strengthen iCARE's applicability in clinical practice." (p23)

And also potential privacy and security concerns related to our framework:

"Regarding privacy, future implementations will be ensured to account for compliance with regulations such as HIPAA and GDPR when incorporating real-world patient data. Furthermore, it is important to acknowledge the security risks associated with the datasets used for model training. Models trained on sensitive private data can be vulnerable to misuse or abuse, particularly in scenarios where adversaries may attempt to extract identifiable information or exploit model behavior. Future work should also consider integrating privacy-preserving techniques such as differential privacy or secure multi-party computation to protect both the data and the individuals represented in it." (p23)

"Lastly, Ensuring fairness in feature selection is crucial, particularly when population representation is imbalanced. iCARE does not explicitly enforce fairness constraints, but its use of locally weighted learners inherently accounts for patient similarity, including demographic factors like race. By computing similarity scores, iCARE can identify whether an incoming patient has sufficiently comparable cases in the dataset. If not, the system can flag them as outliers, prompting caution in interpreting recommendations. Future work could build on this feature to formally incorporate fairness-aware methodologies and improve iCARE's reliability across diverse populations." (p24)

We have also considered the scalability and computational cost of iCARE in more detail. We added a new section to our "*Findings and Interpretation*" titled "*Timing Analysis*" that highlights where iCARE stands in comparison to other approaches in terms of computation time:

"To evaluate the practicality of each feature recommendation approach in real-world scenarios, we conducted a timing analysis using two different datasets: the Early Diabetes Dataset and a synthetic dataset created for controlled experimentation. The primary objective is to assess how the number of patients requiring recommendations and the number of available features affect the computational efficiency of the methods. Importantly, our experimental setup simulates a real-world clinical setting, where patients arrive one at a time and recommendations are made on a per-patient basis. This avoids assumptions of batch processing or the possibility of precomputing feature recommendations. This setup is more realistic because in actual deployments, new patients may update the pool of known cases, invalidating any precomputed global recommendations.

The first part of the experiment investigates how the number of patients requiring feature recommendation affects the runtime. Using the Early Diabetes Dataset, we simulated a scenario where each patient starts with three known features—'Age', 'Gender', and 'Obesity'—and the remaining features are to be recommended. We varied the number of patients (N = 20, 40, 60, 80, 100), ensuring that each recommendation is performed sequentially, without batching. We plotted the result in Figure 13, the left graph, to show the timing relationship. In this setting, we

observed that the Global SHAP method consistently yielded the fastest recommendation times. iCARE, Sequential Forward Selection (SFS), and LASSO required more time, and eGuided was the slowest among the methods tested.

The second part of the experiment focused on how the number of available features for recommendation influences timing. To isolate this variable, we constructed a synthetic dataset comprising 500 samples and 100 randomly generated features, along with an added binary class label. We then varied the number of features available for selection (N = 1, 20, 40, 60), again applying each recommendation method to one patient at a time. As seen in Figure 13, the right graph, Global SHAP and iCARE outperform the other methods. Their runtimes remained relatively stable despite the increasing number of features. eGuided and SFS showed moderate performance with a linear trend, and LASSO demonstrated significantly higher runtimes.

These findings align well with the computational structure of each method. Global SHAP is the most efficient due to its lightweight training process for logistic regression models and calculation of SHAP values, which are both fast and insensitive to sample size and feature dimensionality. iCARE leverages SHAP for personalized recommendations, making it similarly insensitive to feature count, though it incurs additional linear time with the number of patients due to weight calculations for each sample. SFS is slower because it iteratively adds one feature at a time based on model performance, requiring multiple rounds of model training. LASSO also requires retraining and regularization over the feature space, making it computationally expensive. Lastly, eGuided is the most computationally intensive because it identifies the 100 most similar cases, imputes unknown features to generate synthetic samples, and then computes SHAP values on those samples." (p20-21)

We also added an accompanying Figure 13 (response document Figure 2) with the following caption:

"Figure 13: Timing Performance of Feature Recommendation Methods Across Varying Sample Sizes and Feature Dimensions. Runtime comparisons for five feature recommendation methods: Global SHAP, SFS, LASSO, iCARE, and eGuided, under two experimental conditions. The left panel illustrates how the runtime scales with the number of patients (N = 20 to 100) using the Early Diabetes Dataset, where each patient initially has three known features. The right panel displays runtime behavior as the number of available features increases (1 to 60) using a synthetic dataset of 500 samples and 100 total features." (p21-22)

3. **Compare with Other State-of-the-Art Methods:**
- Include a comparison with other personalized feature selection methods, particularly those that have been applied in clinical or medical contexts.

- Discuss how iCARE performance compares to these methods and what unique advantages it offers.

Thank you for pointing out the need for a more comprehensive comparison with other state-of-the-art personalized feature selection methods. We acknowledge that the original manuscript included a limited set of comparative baselines. While the eGuided framework remains the most directly comparable state-of-the-art method, particularly due to its use of SHAP values for guided feature selection, we agree that additional comparisons are essential for situating iCARE within the broader landscape. In response, and alignment with a recommendation from another reviewer, we have now incorporated results from commonly used feature selection methods such as Lasso and Sequential Feature Selection (SFS). We provided the following text in the Results section titled "*Comparison with Other Frameworks*" to report these new findings:

"We also provide comparisons with other global feature selection methods, which are SHAP-based (Global), SFS, and Lasso. As shown in Figure 12, iCARE achieved a 6% higher ROC-AUC score on average in the Early Diabetes Dataset and a 12.1% higher ROC-AUC score on average in the Heart Disease Dataset compared to Eguided. Over the global feature selection methods, iCARE showed around the same increase in performance." (p18)

and the following text to the figure titled "Figure 12: Performance Comparison of Feature Selection Methods Across Three Datasets." (response document Figure 1) to adjust for the new methods that were added:

"Figure 12: Performance Comparison of Feature Selection Methods Across Three Datasets. This figure presents a comparative evaluation of AUC-ROC and Accuracy across different feature selection approaches on three real-world datasets: (a) Early Diabetes, (b) Heart Failure, and (c) Heart Disease. The graphs illustrate the performance of five feature selection methods, which are Global (blue), SLS (orange), LASSO (green), iCARE (red), and eGuided (purple), across varying feature subsets. The x-axis represents the number of selected features, while the y-axis shows the corresponding AUC-ROC and Accuracy scores, providing insights into the effectiveness of each method in optimizing predictive performance." (p20)

4. **Clarify SHAP Value Calculation:**
- Provide a more detailed explanation of how SHAP values are calculated and interpreted within the iCARE framework, particularly in the context of personalized feature selection.

We appreciate the suggestion and have added the following explanation to the Methods section under "*Framework Architecture*" to clarify how SHAP values are calculated and interpreted within the iCARE framework:

“SHAP treats each feature like a “player” in a game and calculates how much it helps the model make its prediction when it joins the team of features. It does this by computing the average change in the prediction when that feature is added to all possible combinations of the other features. In iCARE, we use a SHAP linear explainer to explain our weighted logistic regression model. For each patient, we calculate SHAP values to see how strongly each feature pushes the prediction higher or lower. By averaging these contributions, we identify which features consistently have the biggest impact.” (p4)

5. **Expand on Future Directions:**
- Discuss potential applications of the iCARE framework beyond clinical assessments, such as in predictive maintenance, financial forecasting, or other fields where personalized feature selection is valuable.
- Explore how the framework could be integrated with other machine learning algorithms or extended to address more complex clinical scenarios.

Thank you for the thoughtful suggestion regarding potential applications beyond clinical assessments. In response, we have expanded the Conclusion section to more clearly articulate the model-agnostic nature of the iCARE framework. We now emphasize that while logistic regression was used in this study, iCARE can be readily integrated with any machine learning or deep learning model designed for classification tasks. Additionally, we highlight several potential use cases in other domains where personalized feature selection could be valuable, such as predictive maintenance, financial forecasting, and personalized education systems. Here’s the edited passage in the Conclusion section to incorporate the suggestion:

“Although personalization might not be needed in all cases, iCARE provides a flexible framework that can be applied using other machine learning algorithms. While we implemented iCARE with logistic regression in this study, the underlying framework is model-agnostic and can be adapted to work with any machine learning or deep learning model designed for classification tasks. We believe that with further testing iCARE can be applied to other domains where personalized feature recommendation can improve decision-making, such as predictive maintenance in industrial systems, financial risk assessment, or personalized learning systems in education.” (p24)

Conclusion:
The iCARE framework represents a significant contribution to the field of personalized medicine and clinical assessments. With the suggested revisions, particularly in terms of discussing related

work and expanding on the framework's limitations and future directions, the manuscript will be strengthened and provide a more comprehensive contribution to the literature.
